# Supplementary material for: Direct Neuronal Reprogramming of Common Marmoset Fibroblasts by ASCL1, microRNA-9/9*, and microRNA-124 Overexpression
Source: Cells. 2020 Dec 22;10(1):6. doi: 10.3390/cells10010006 (PMC7822173; doi:10.3390/cells10010006)
Supplement: Supplementary file 1 [file cells-10-00006-s001.zip › Supplementary Materials/Supplementary files.pptx]

## Slide 1
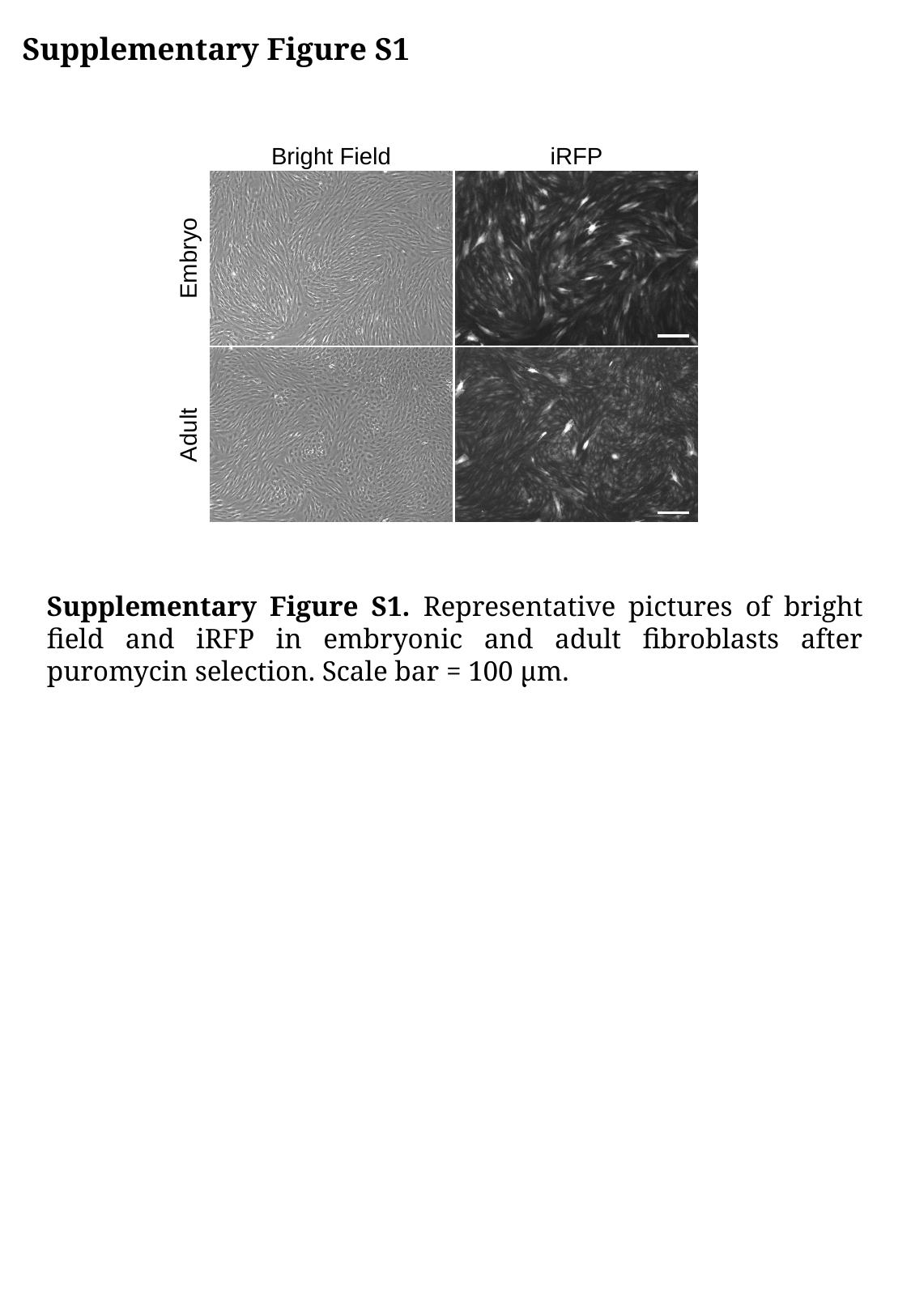

Supplementary Figure S1
Bright Field
iRFP
Embryo
Adult
Supplementary Figure S1. Representative pictures of bright field and iRFP in embryonic and adult fibroblasts after puromycin selection. Scale bar = 100 µm.

## Slide 2
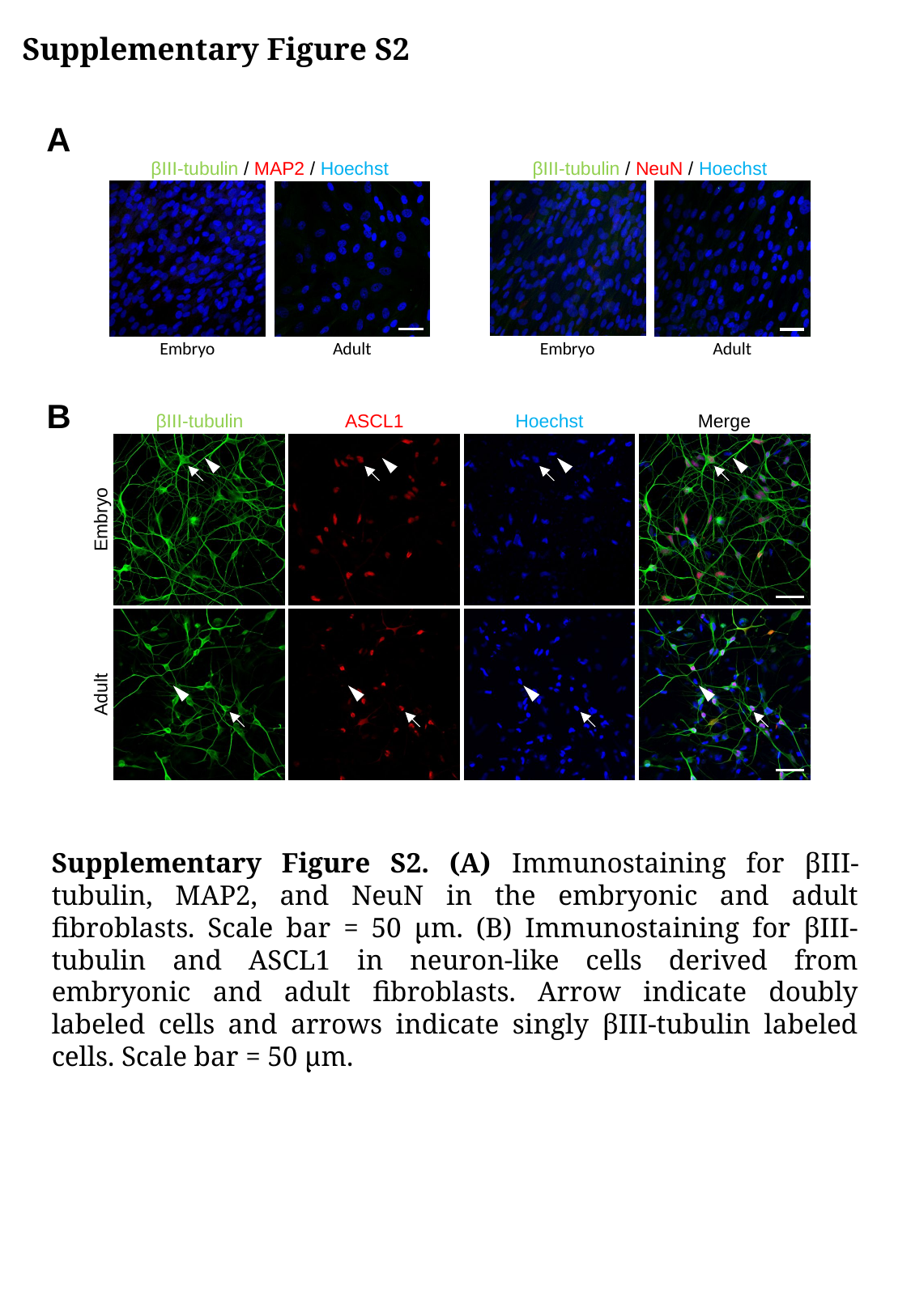

Supplementary Figure S2
A
βIII-tubulin / NeuN / Hoechst
βIII-tubulin / MAP2 / Hoechst
Embryo
Adult
Embryo
Adult
B
βIII-tubulin
ASCL1
Hoechst
Merge
Embryo
Adult
Supplementary Figure S2. (A) Immunostaining for βIII-tubulin, MAP2, and NeuN in the embryonic and adult fibroblasts. Scale bar = 50 µm. (B) Immunostaining for βIII-tubulin and ASCL1 in neuron-like cells derived from embryonic and adult fibroblasts. Arrow indicate doubly labeled cells and arrows indicate singly βIII-tubulin labeled cells. Scale bar = 50 µm.

## Slide 3
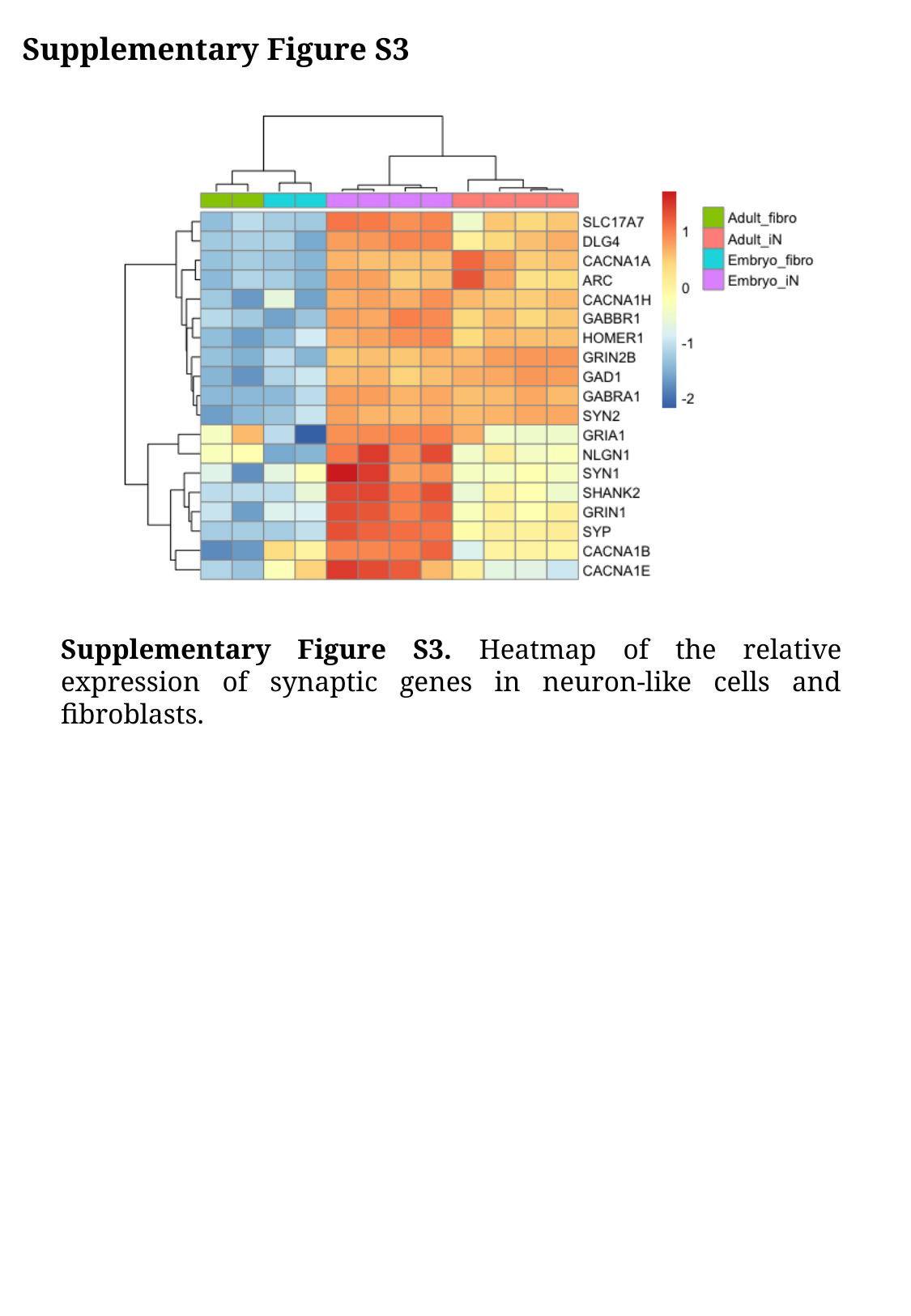

Supplementary Figure S3
Supplementary Figure S3. Heatmap of the relative expression of synaptic genes in neuron-like cells and fibroblasts.

## Slide 4
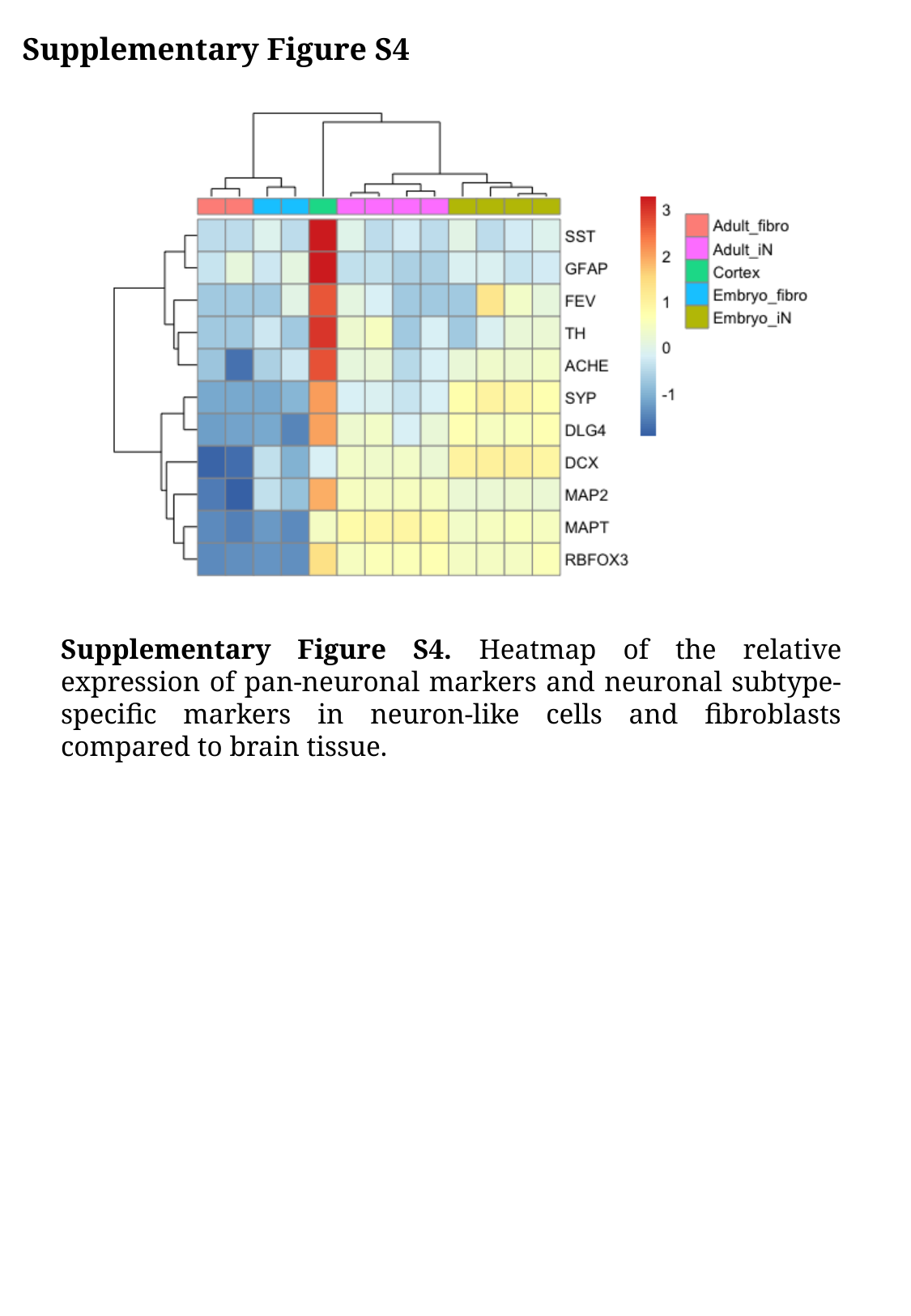

Supplementary Figure S4
Supplementary Figure S4. Heatmap of the relative expression of pan-neuronal markers and neuronal subtype-specific markers in neuron-like cells and fibroblasts compared to brain tissue.

## Slide 5
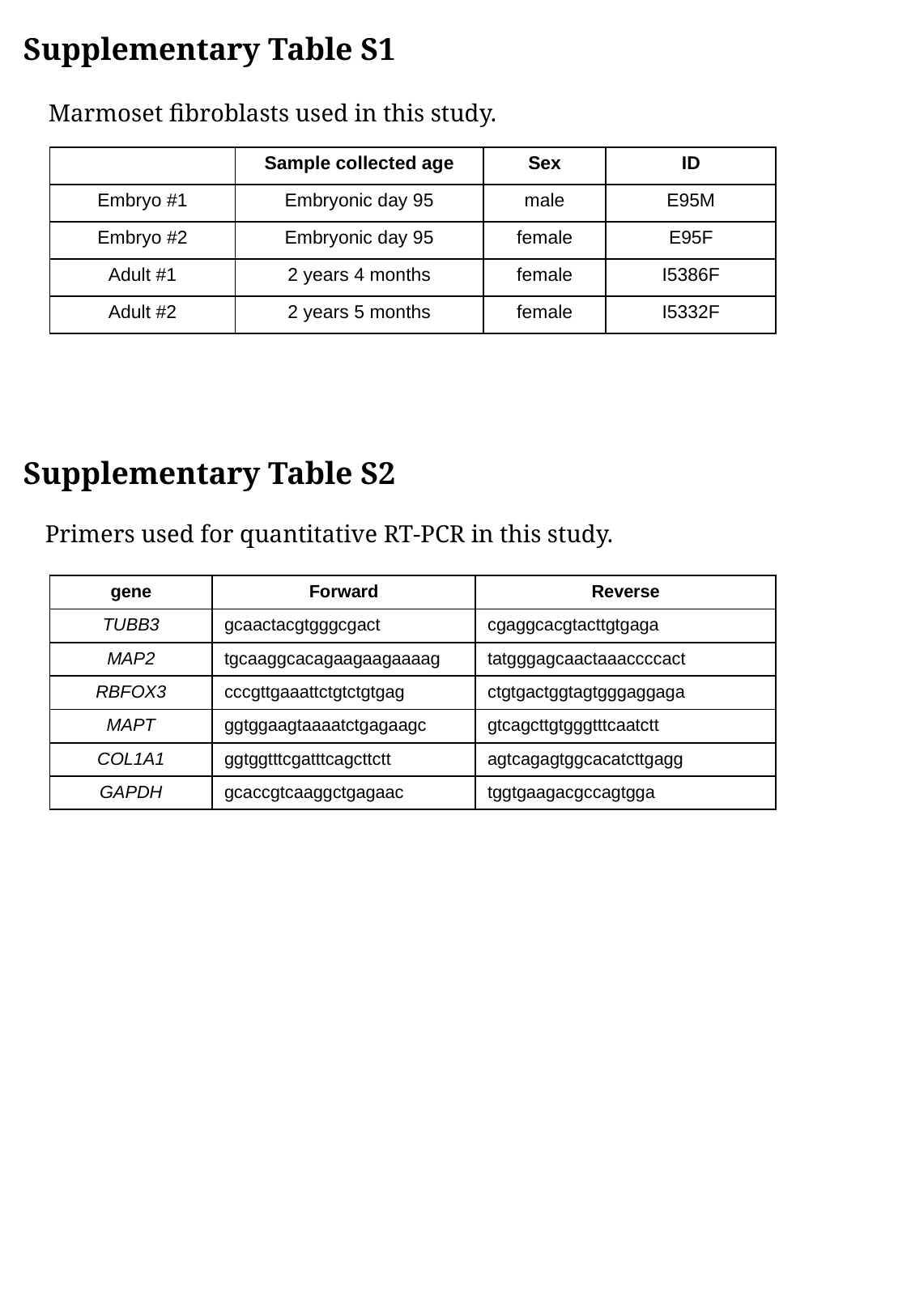

Supplementary Table S1
Marmoset fibroblasts used in this study.
| | Sample collected age | Sex | ID |
| --- | --- | --- | --- |
| Embryo #1 | Embryonic day 95 | male | E95M |
| Embryo #2 | Embryonic day 95 | female | E95F |
| Adult #1 | 2 years 4 months | female | I5386F |
| Adult #2 | 2 years 5 months | female | I5332F |
Supplementary Table S2
Primers used for quantitative RT-PCR in this study.
| gene | Forward | Reverse |
| --- | --- | --- |
| TUBB3 | gcaactacgtgggcgact | cgaggcacgtacttgtgaga |
| MAP2 | tgcaaggcacagaagaagaaaag | tatgggagcaactaaaccccact |
| RBFOX3 | cccgttgaaattctgtctgtgag | ctgtgactggtagtgggaggaga |
| MAPT | ggtggaagtaaaatctgagaagc | gtcagcttgtgggtttcaatctt |
| COL1A1 | ggtggtttcgatttcagcttctt | agtcagagtggcacatcttgagg |
| GAPDH | gcaccgtcaaggctgagaac | tggtgaagacgccagtgga |

## Slide 6
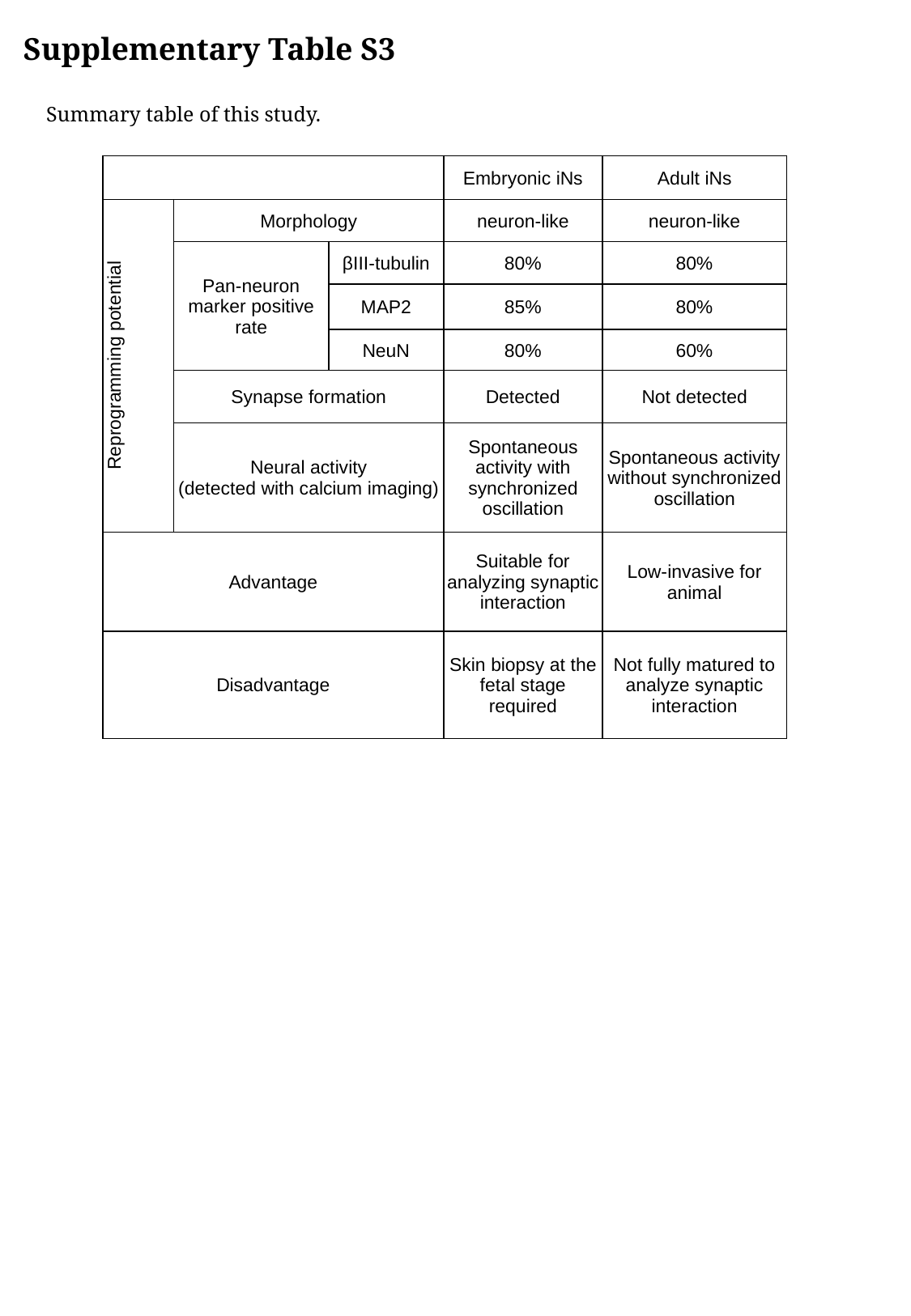

Supplementary Table S3
Summary table of this study.
| | | | Embryonic iNs | Adult iNs |
| --- | --- | --- | --- | --- |
| Reprogramming potential | Morphology | | neuron-like | neuron-like |
| | Pan-neuron marker positive rate | βIII-tubulin | 80% | 80% |
| | | MAP2 | 85% | 80% |
| | | NeuN | 80% | 60% |
| | Synapse formation | | Detected | Not detected |
| | Neural activity (detected with calcium imaging) | | Spontaneous activity with synchronized oscillation | Spontaneous activity without synchronized oscillation |
| Advantage | | | Suitable for analyzing synaptic interaction | Low-invasive for animal |
| Disadvantage | | | Skin biopsy at the fetal stage required | Not fully matured to analyze synaptic interaction |

## Slide 7
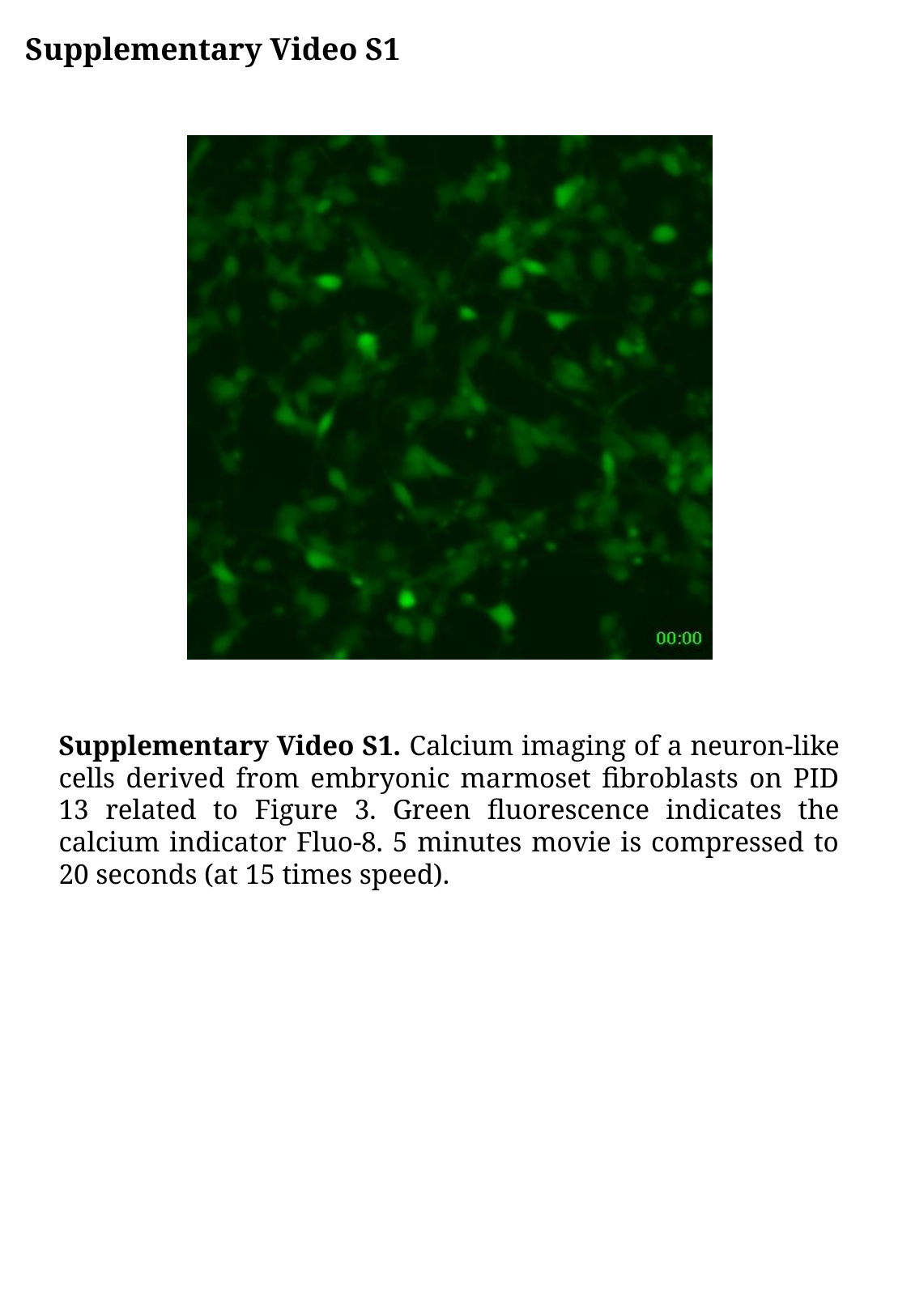

Supplementary Video S1
Supplementary Video S1. Calcium imaging of a neuron-like cells derived from embryonic marmoset fibroblasts on PID 13 related to Figure 3. Green fluorescence indicates the calcium indicator Fluo-8. 5 minutes movie is compressed to 20 seconds (at 15 times speed).

## Slide 8
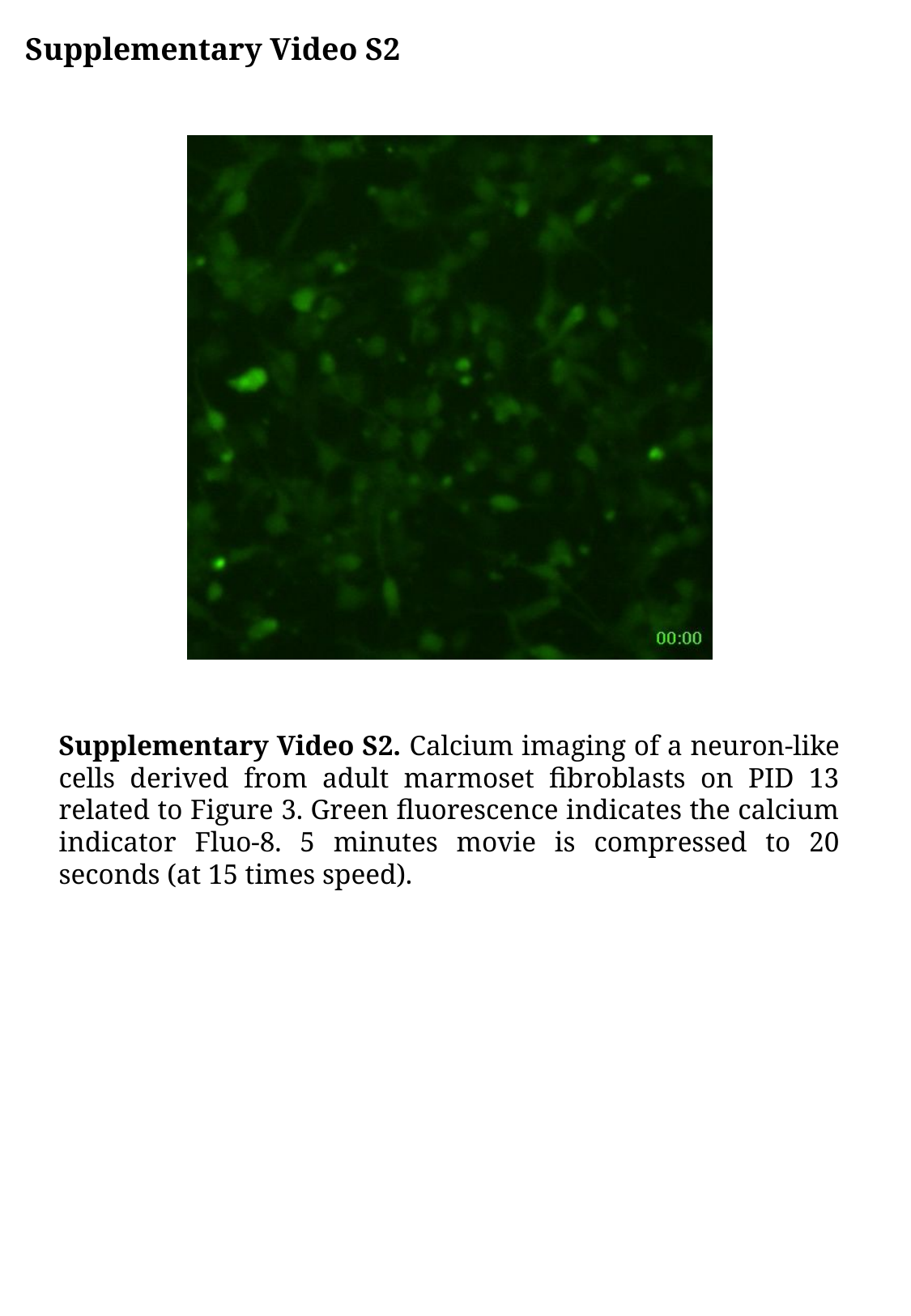

Supplementary Video S2
Supplementary Video S2. Calcium imaging of a neuron-like cells derived from adult marmoset fibroblasts on PID 13 related to Figure 3. Green fluorescence indicates the calcium indicator Fluo-8. 5 minutes movie is compressed to 20 seconds (at 15 times speed).
